# Bilateral Testicular Torsion: A Systematic Review of Case Reports

**DOI:** 10.7759/cureus.38861

**Published:** 2023-05-10

**Authors:** Akram Bokhari, Hadi Aldarwish, Turki Alharbi, Yasir Alrashidi, Abdulrahman Alharbi, Lafi Alsulami

**Affiliations:** 1 Urology, University of Hail College of Medicine, Hail, SAU; 2 Urology, Miami Cancer Institute, Florida, USA; 3 Surgery, University of Hail College of Medicine, Hail, SAU

**Keywords:** orchidopexy, bilateral testicular torsion, torsion, spermatic cord, testis

## Abstract

*Bilateral testicular torsion* is a rare but serious condition characterized by twisting both testicles around their respective spermatic cords, leading to reduced blood flow and potential loss of the testicles. Treatment of this condition may involve surgical detorsion of the affected testicles and fixation to prevent recurrence and, in some cases, removal of severely damaged testicles. In April 2023, a systematic review of case reports was performed to examine the presentation, clinical features, diagnostic process, and management of bilateral testicular torsion. Our search encompassed the following databases: PubMed, ScienceDirect, and Google Scholar. Out of 340 studies, only eight cases met our criteria. However, this review discusses bilateral testicular torsion symptoms, investigation, and outcome.

## Introduction and background

*Bilateral testicular torsion* is a rare but serious condition characterized by twisting both testicles around their respective spermatic cords, leading to reduced blood flow and potential loss of the testicles [1]. This condition often presents with acute scrotal pain, swelling, and tenderness and requires urgent surgical intervention to prevent permanent damage [2].

The incidence of bilateral testicular torsion is estimated to be less than 2% of all cases of testicular torsion, with most cases occurring in neonates males. The exact causes of bilateral testicular torsion are not fully understood, but it has been associated with anatomical abnormalities, trauma, and certain activities that involve sudden, forceful movements of the lower body [3].

Diagnosis of bilateral testicular torsion is typically made through physical examination, imaging tests such as ultrasound, and measurement of blood flow to the affected testicles [4]. Management involves surgical detorsion of the affected testicles and fixation to prevent recurrence and, in some cases, removal of severely damaged testicles [5]. This article aims to enhance understanding of the clinical presentation, diagnostic process, and outcome of bilateral testicular torsion.

## Review

Materials and methods

Following the Preferred Reporting Items for Systematic Reviews and Meta-Analyses (PRISMA) guidelines [6], we conducted a systematic review of case reports to identify and analyze cases of bilateral testicular torsion. Our search encompassed the following databases: PubMed, ScienceDirect, and Google Scholar.

Search Strategy

To identify relevant articles on bilateral testicular torsion, we conducted a search strategy on PubMed. Our approach involved identifying keywords related to the condition and combining them with medical subject headings (MeSH). Using the Medical Literature Analysis and Retrieval System Online (MEDLINE), we developed a final search strategy that integrated Boolean operators as follows: "bilateral testicular torsion OR spermatic cord torsion OR bilateral torsion of testes".

To ensure that our study was focused on the specific area of interest, we conducted searches on ScienceDirect and Google Scholar using the keywords "bilateral testicular torsion" to find relevant articles.

Inclusion and Exclusion Criteria

During the data extraction process, we utilized the criteria outlined in Table 1 to comprehensively examine cases related to bilateral testicular torsion and its current management.

**Table 1:** List of Inclusion and Exclusion Criteria

Criterion	Description
First criterion	Fields of study related to medicine.
Second criterion	Selection of case reports only.
Third criterion	Sorting especially for free full-text articles.
Fourth criterion	English-language peer-reviewed studies.

Results

**Figure 1:**
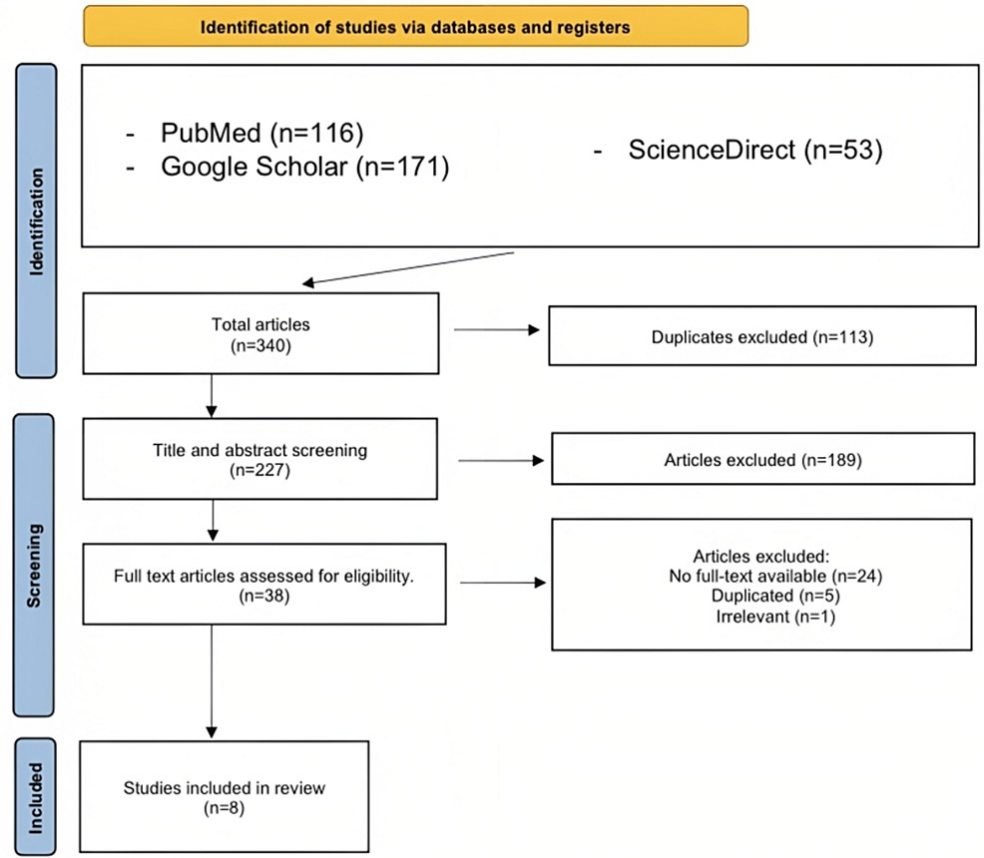
PRISMA Flowchart PRISMA: Preferred Reporting Items for Systematic Reviews and Meta-Analyses References- [7-14]

Utilizing the above methods, our search across multiple databases yielded 340 publications. To remove duplicate publications from the initial investigation, we used EndNote (Clarivate, London, United Kingdom) and manual comparison, which led to the removal of 113 duplicate publications, leaving us with 227 articles for further analysis. We then thoroughly screened the titles and abstracts of these articles to determine their relevance to our research topic. Finally, we evaluated the quality of the remaining eight studies using the Joanna Briggs Institute (JBI) Critical Appraisal Checklist for Case Reports (figure 1). Patient demographics, country of origin, clinical presentation, management plan, and prognosis for the eight cases are summarized in table 2.

**Table 2:** Overview of selected cases. References- [7-14]

Author	Year	Patient demographics	Country	Presentation	Management	Prognosis
Lorenzo et al. [7]	2018	15-year-old	Espanha	Left hemiscrotal pain, tenderness, and swelling lasted for 48 hours	Right testis fixation and left Orchiectomy	1 year after surgery, the patient’s right testicle was normal in size, echogenicity, and vascularization
Beliaev et al. [8]	2013	16–year-old	New Zealand	3-hour history of left hemi-scrotal pain, nausea, and a single episode of vomiting	Untwisting of both testicles and orchidopexies.	NA
Dhua et al. [9]	2014	3-day-old	India	A preterm neonate with complaints of bilateral scrotal swelling was observed on 3rd day of life.	Left orchiectomy with the right testis untwisted	At follow-up after 2 months, the right testis showed signs of late atrophy, 2 months later, the patient developed subcutaneous gangrene of the umbilical region, but the parents refused to admit the patient, then he died at home due to sepsis
Lee et al. [10]	2002	4-day-old	Korea	A full-term newborn with right scrotal swelling since birth was transferred to a specialized facility for evaluation and treatment on the 4th day after birth.	Untwisting of both testicles	At the age of 2 months, both testicles were palpable but smaller than normal
Osada et al. [11]	1985	12-year-old	Japan	Bilaterally painfully enlarged testes for the past 60 hours	Bilateral orchidopexy	After 1 year, both testes were palpable but smaller than normal size, and the testosterone levels were markedly low
Sommer-Jörgensen et al. [12]	2020	newborn	Switzerland	Discoloration and signiﬁcantly painful swelling of the scrotum 4 hours after birth	Detorsion and bilateral orchidopexy	At 2,6 and 12 months old follow-up, both testes were palpable and within normal size. Endocrine investigation at 2 months showed normal hormonal levels
Clarke et al. [13]	2018	newborn	UK	Macrocosmic male infant born to a diabetic mother had bilaterally enlarged and firm testes. US was performed on day 3 of life and showed bilateral testicular torsion	Conservatively	NA
Pogorelić et al. [14]	2018	newborn	Croatia	Diffuse swelling and redness of the scrotum, more evident on the right side, hard and tender on palpation.	Right orchiectomy with left testicle fixation	Follow-up at 6 months showed left testicle atrophy and very low hormonal assay.

Discussion

*Bilateral testicular torsion* is a rare urological emergency characterized by twisting both testes along their axis, resulting in compromised blood flow and potentially leading to testicular ischemia and necrosis [15]. Although the exact incidence of bilateral testicular torsion is unknown, it is estimated to be less than 2% of all cases of testicular torsion. It is most observed in neonates because of their incompletely developed scrotal attachment [7,11].

Testicular torsion can be categorized into two types: intravaginal and extravaginal. In the perinatal period, extravaginal torsion is more commonly observed due to incomplete development of the attachments between the tunica vaginalis and the scrotal wall. However, as the child grows and the attachments become stronger, the occurrence of extravaginal torsion declines. On the other hand, intravaginal torsion occurs during puberty due to anatomic abnormalities within the tunica vaginalis, such as the Bell-Clapper deformity, and during periods of testicular growth [10,13].

Not surprisingly, bilateral testicular torsion shares many clinical features with unilateral testicular torsion, including the presence of acute-onset bilateral scrotal pain, swelling, and tenderness. However, the absence of the classic "bell-clapper" deformity, a common finding in unilateral torsion, may make diagnosing bilateral testicular torsion more challenging [1,16]. Other differential diagnoses, such as epididymitis, testicular trauma, or tumors, should also be considered [17].

Moreover, the diagnosis of bilateral testicular torsion is typically made based on clinical suspicion and confirmed by imaging studies, such as scrotal ultrasound; the finding can range from the normal testis to enlarge testis due to edema from venous occlusion [18]. However, the sensitivity of ultrasound in bilateral testicular torsion cases may be lower than in cases of unilateral torsion, and a high index of suspicion is required for accurate diagnosis [8]. The literature suggests that combining radiographic features with Doppler ultrasound can result in a sensitivity range of 88.9% to 100% and a specificity range of 97.9% to 98.8% for detecting testicular torsion. [19,20].

The management of bilateral testicular torsion is similar to that of unilateral torsion and involves an urgent surgical intervention to untwist the testes and restore blood flow [21]. In cases where testicular viability is in doubt, bilateral orchiectomy may be necessary to prevent further complications such as sepsis and abscess formation [22]. However, in our literature, all cases were managed surgically by untwisting testis and fixation except one, which was managed conservatively. 

The potential long-term complications of bilateral testicular torsion include testicular atrophy and infertility, which may require further management, such as assisted reproductive techniques. Patients with bilateral testicular torsion should be closely followed up to monitor these potential complications and ensure appropriate management [12,13]. In our review cases, follow-ups were done to check the size of the testis, echogenicity, vascularization, and testosterone level.

The duration of symptoms prior to detorsion and the degree of twisting is crucial prognostic factors determining the extent of testicular damage and its potential for salvageability [7]. The viability of the testis starts to deteriorate significantly after six hours of symptom onset [23]. In addition, a study conducted by Tryfonas et al. revealed that if torsion exceeds 360° and symptoms persist for more than 24 hours, the testes could be absent or severely atrophied [24]. Hence, it is essential for clinicians to promptly recognize this surgical emergency and seek urgent consultation with a urologist if they suspect testicular torsion.

Limitations

The main limitation of this review was its reliance on case reports, which lacked generalizability. Moreover, including cohort studies in a review article would provide a more accurate understanding of bilateral testicular torsion's presentation, management, and prognosis.

## Conclusions

*Bilateral testicular torsion* is a severe but rare urological emergency that requires prompt diagnosis and management. The prevalence of this condition is less than 2% of all cases of testicular torsion, and it is most common in neonates. Although bilateral testicular torsion shares many clinical features with unilateral torsion, including scrotal pain, swelling, and tenderness. Moreover, the absence of the classic "bell-clapper" deformity may make diagnosis more challenging. A high index of suspicion, careful evaluation, and appropriate imaging studies are necessary for accurate diagnosis and timely intervention. Combining radiographic features with Doppler ultrasound can result in high sensitivity and specificity for detecting testicular torsion. The management of bilateral testicular torsion is similar to that of unilateral torsion and involves urgent surgical intervention and orchiopexy. The viability of the testis starts to deteriorate significantly after six hours of symptom onset. So, surgical emergencies and seeking urgent consultation with a urologist are essential. The potential long-term complications highlight the need for close follow-up and appropriate management to minimize morbidity and maximize fertility outcomes. Finally, further research is needed to better understand bilateral testicular torsion's pathogenesis and risk factors.
